# Behavioural compass: animal behaviour recognition using magnetometers

**DOI:** 10.1186/s40462-019-0172-6

**Published:** 2019-08-27

**Authors:** Pritish Chakravarty, Maiki Maalberg, Gabriele Cozzi, Arpat Ozgul, Kamiar Aminian

**Affiliations:** 1 School of Engineering, Ecole Polytechnique Fédérale de Lausanne, Lausanne, Switzerland; 2 School of Information Technologies, Tallinn University of Technology, Tallinn, Estonia; 3 Department of Evolutionary Biology and Environmental Studies, University of Zurich, Zurich, Switzerland; 4 Kalahari Research Centre, Kuruman River Reserve, Van Zylsrus, 8467 South Africa

**Keywords:** Magnetometer, Behaviour recognition, Biomechanics, Machine learning, Angular velocity, Earth’s magnetic field, Accelerometer, Meerkats

## Abstract

**Background:**

Animal-borne data loggers today often house several sensors recording simultaneously at high frequency. This offers opportunities to gain fine-scale insights into behaviour from individual-sensor as well as integrated multi-sensor data. In the context of behaviour recognition, even though accelerometers have been used extensively, magnetometers have recently been shown to detect specific behaviours that accelerometers miss. The prevalent constraint of limited training data necessitates the importance of identifying behaviours with high robustness to data from new individuals, and may require fusing data from both these sensors. However, no study yet has developed an end-to-end approach to recognise common animal behaviours such as foraging, locomotion, and resting from magnetometer data in a common classification framework capable of accommodating and comparing data from both sensors.

**Methods:**

We address this by first leveraging magnetometers’ similarity to accelerometers to develop biomechanical descriptors of movement: we use the static component given by sensor tilt with respect to Earth’s local magnetic field to estimate posture, and the dynamic component given by change in sensor tilt with time to characterise movement intensity and periodicity. We use these descriptors within an existing hybrid scheme that combines biomechanics and machine learning to recognise behaviour. We showcase the utility of our method on triaxial magnetometer data collected on ten wild Kalahari meerkats (*Suricata suricatta*), with annotated video recordings of each individual serving as groundtruth. Finally, we compare our results with accelerometer-based behaviour recognition.

**Results:**

The overall recognition accuracy of > 94% obtained with magnetometer data was found to be comparable to that achieved using accelerometer data. Interestingly, higher robustness to inter-individual variability in dynamic behaviour was achieved with the magnetometer, while the accelerometer was better at estimating posture.

**Conclusions:**

Magnetometers were found to accurately identify common behaviours, and were particularly robust to dynamic behaviour recognition. The use of biomechanical considerations to summarise magnetometer data makes the hybrid scheme capable of accommodating data from either or both sensors within the same framework according to each sensor’s strengths. This provides future studies with a method to assess the added benefit of using magnetometers for behaviour recognition.

**Electronic supplementary material:**

The online version of this article (10.1186/s40462-019-0172-6) contains supplementary material, which is available to authorized users.

## Background

Behaviour is a central component of any animal’s life and the result of important biotic and abiotic interactions. Its accurate description is therefore crucial for a full appreciation of an animal’s biology. Small, light-weight animal-borne data loggers have proved to be indispensable as they bypass the logistical difficulties of directly observing animals and enable data to be collected on an animal as it goes about its daily life in its natural environment [1]. Data loggers today can often house several sensors (e.g. [2, 3]), each measuring different physical quantities such as acceleration, magnetic field intensity, angular velocity, light level, and depth. Simultaneously recorded high-frequency multi-sensor data offer the opportunity to gain fine-scale insights into behaviour by leveraging information not only from individual data streams, but also by fusing data from multiple sensors.

In the context of animal behaviour recognition, triaxial accelerometers [4] and magnetometers [5] have both been used to identify movement patterns in animals. Though accelerometers have by far been used more extensively (e.g. [6–10]), it has recently been shown that magnetometers can better resolve certain low-acceleration behaviours of biological importance, e.g. thermal soaring in Andean condors (*Vultur gryphus*) [11]. In fact, a recent comparison of accelerometers and magnetometers has demonstrated that there can be quantifiably large differences in recognition capability between the two sensors for certain specific behaviours [5]. Future behaviour recognition algorithms may thus seek to leverage the complementarity of these two sensors by fusing data from both sensors within a single classification framework. However, it is not known how recognition capability differs between the two sensors for the case of common animal behaviours such as foraging, locomotion, and resting.

One of the reasons for the success of accelerometers in recognising animal behaviour may be their ability to measure both static tilt with respect to Earth’s gravity vector as well as dynamic acceleration resulting from animal motion. Despite the numerous advantages of the accelerometer, however, the sensor has some inherent limitations that may render it unsuitable for use in certain situations. Firstly, during dynamic movements, the accelerometer is sensitive to both body segment tilt and dynamic acceleration due to motion. Dynamic acceleration interferes with the change of tilt, and the two cannot be separated. In extreme cases, such as when an animal is ‘pulling g’ [5] or in freefall, the accelerometer cannot be used to measure tilt because the total measured acceleration approaches zero. Secondly, for the same activity, signal magnitudes vary greatly depending upon sensor location on the body [12]. This may be problematic for fine-scale estimation of behavioural parameters. For instance, in human accelerometer-based pedometer applications, the accuracy of step counting changes if the pedometer is attached to any location other than the waist [13]. Thirdly, accelerometers may not be well-suited to detection and characterisation of dynamic behaviours involving slow, especially rotation-based, movement [11].

Magnetometers bear surprising similarities to accelerometers: they can measure a static component through inclination with respect to Earth’s magnetic field as well as a dynamic component corresponding to changes in sensor inclination over time. The static component has been used extensively to obtain animal heading and perform dead-reckoning (e.g. [14–16]). The resulting movement paths have been used to, for instance, quantify differences between straight-line and tortuous-path travel to infer underlying behaviour [17, 18], and understand animals’ sense of orientation [19]. The dynamic component of the magnetometer has been used to extract metrics describing angular velocity for human wearable sensing applications [20, 21]. In spite of these similarities, magnetometers are not prone to the problems highlighted above for accelerometers. Firstly, the magnetometer directly measures sensor tilt. The dynamic component is not mixed with the static component of the signal, and may be obtained by differentiating the signal with respect to time [20]. Note, however, that when the axis of rotation happens to align exactly with the local magnetic field line – an unlikely scenario over extended time – the dynamic component will be zero [5]. The equivalent operation for the accelerometer (i.e. integration with respect to time) does not directly provide velocity because of the need to resolve the constant of integration through knowledge of initial or final velocity from a different source. Secondly, since it is likely that a wild animal’s natural habitat will be far from man-made sources of magnetic field disturbances, the signal magnitude will be the same regardless of activity type or sensor location on the body. Note, however, that the presence of magnetic field disturbances might preclude comparison of signal-derived metrics between different locations. Thirdly, magnetometers have been shown to be capable of resolving behaviours that are not easily discerned using accelerometers, such as thermal soaring in Himalayan griffon vultures (*Gyps himalayensis*) [5]. Despite the magnetometer’s potential for behaviour telemetry, there is a lack of an end-to-end method for identifying common animal behaviours from magnetometer data.

Here, we demonstrate that biomechanically relevant features describing posture, movement intensity, and periodicity can be derived from static and dynamic components of recorded magnetometer data. These can be combined with an existing framework (based on acceleration data, [10]) that combines biomechanics and machine learning to assign accelerometer signals into behavioural categories. We showcase the application of these principles for data collected on wild meerkats (*Suricata suricatta*), a social foraging, < 1 kg carnivore inhabiting the Kalahari and Namib deserts of Southern Africa [22], where the classification of their main activities such as vigilance, foraging, resting, and running, is essential for characterising their individual and social behaviour. We provide a comparison of magnetometer-based behaviour recognition performance with the accelerometer-based one, discuss the strengths and weaknesses of the magnetometer as a standalone sensor for behaviour recognition, and discuss possibilities for fusing data from both sensors to achieve more accurate and robust behaviour recognition.

## Methods

### Deriving biomechanical descriptors of movement using magnetometer data

In a recent study on behaviour recognition using accelerometers [10], posture, movement intensity, and periodicity were used as biomechanical descriptors of static and dynamic behaviours.

**Fig. 1:**
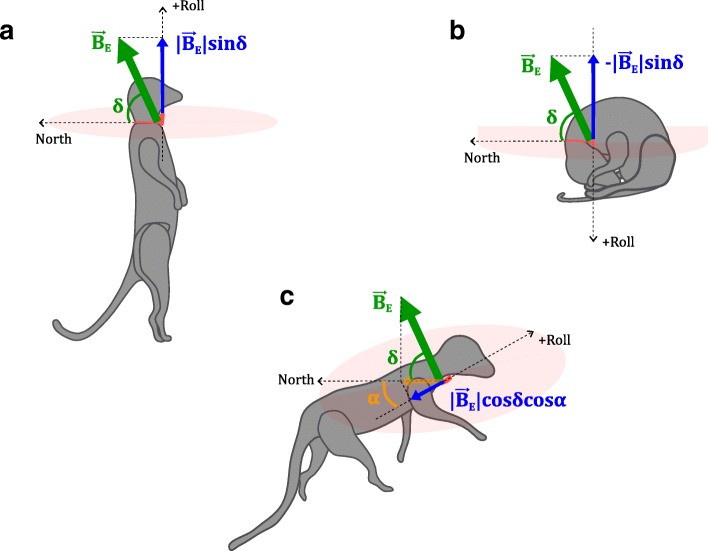
Using magnetometer data to distinguish between different meerkat postures. The Earth’s magnetic field ${\overrightarrow{B}}_E$ (green arrows) inclined at a dip angle of *δ* with respect to the horizontal plane (salmon-pink disk) subtends components (blue arrows) equal in magnitude and opposite in sign along the collar sensor’s (in red) roll axis during (**a**) vigilance, and (**b**) curled-up resting, demonstrated in the simplified case when the roll axis is perfectly aligned with the local vertical direction. When the roll axis lies in the horizontal plane, as shown in (**c**), the measured component of ${\overrightarrow{B}}_E$ is further affected by the possibly arbitrary azimuthal angle *α*

Behaviour separation using posture estimated from magnetometer data can be achieved when a given axis of the sensor aligns in two opposite directions along the vertical axis for the two static behaviours to be separated (Fig.1). [10] used the accelerometer’s surge axis, which corresponds to the same direction as that of the magnetometer’s roll axis in the present study, for quantifying posture since values along this axis were least susceptible to changes caused by possible rotations of the collar around the axis of the meerkat’s cylindrical neck. Let ${\overrightarrow{B}}_E$ be the local magnetic field vector with dip angle *δ* at the sensor location. During meerkat vigilance (Fig. 1a), in an idealised case, the roll axis would point directly upwards, perpendicular to the horizontal plane (the latter shown as a salmon-pink disk), and the sensor’s roll axis would measure $\left|{\overrightarrow{B}}_E\right| sin\delta$. During curled-up resting (Fig. 1b), on the other hand, the roll axis would point downwards, perpendicular to the horizontal plane, and the sensor’s roll axis would measure $-\left|{\overrightarrow{B}}_E\right| sin\delta$. We hypothesised that this polarity (positive and negative value of $\left|{\overrightarrow{B}}_E\right| sin\delta$) would enable discrimination of the two static behaviours, vigilance and curled-up resting. When the roll axis lies in the horizontal plane (Fig. 1c), however, the measurement along the roll axis of the projection of ${\overrightarrow{B}}_E$ onto the horizontal plane, $\left|{\overrightarrow{B}}_E\right| cos\delta$, would be affected by the azimuthal orientation of the animal (angle *α* between the direction faced with respect to magnetic North in the horizontal plane), and the measured value would now be $\left|{\overrightarrow{B}}_E\right| cos\delta cos\alpha$. Since values of *α* may vary arbitrarily between 0° and 360°, the roll axis would record measurements in the range of [$-\left|{\overrightarrow{B}}_E\right| cos\delta$, $\left|{\overrightarrow{B}}_E\right| cos\delta$] when it lies in the horizontal plane. Thus, static behaviours such as belly-flat resting as well as dynamic behaviours such as foraging and running may be difficult to separate only on the basis of posture, since the sensor’s roll axis can be oriented arbitrarily with respect to the North direction.

In carrying out dynamic activities such as running, the collar sensor would follow in the wake of movements made by the torso and neck as the animal heaves and sways, and rotate around the animal’s neck. Thus, the magnetometer’s axes would continuously change orientation with respect to the local magnetic field lines. The more intense the bodily movement is, the faster the sensor would change orientation with respect to the local field lines. For instance, large oscillations in triaxial magnetometer signals recorded during cheetah (*Acinonyx jubatus*) running behaviour have previously been reported [2]. If this motion is periodic, the change in sensor orientation will also be periodic. Thus, the magnitude of change in recorded signal values could be used as an indicator for the intensity of movement, and help distinguish between static and dynamic behaviours. Further, the periodicity of the rate of change in recorded signal values could be used to distinguish between the dynamic behaviours: for meerkats, running has been shown to be highly periodic, and foraging to be relatively aperiodic [10]. Measures of both intensity and periodicity may be characterised either by computing the amount of variation in the recorded signal itself, through measures such as standard deviation, or by computing the amount of variation in the time-differentiated signal.

### Data collection and groundtruthing

**Fig. 2:**
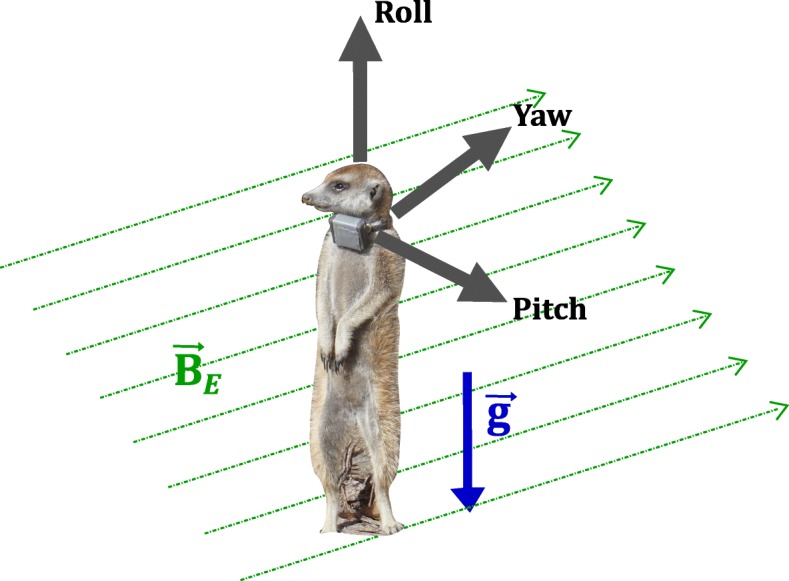
Meerkat with collar, axes, and Earth’s fields. The orientation of the axes of the triaxial magnetometer fixed to a collar on the meerkat along with the directions of two of Earth’s naturally occurring fields: Earth’s magnetic field ${\overrightarrow{B}}_E$ pointing towards the magnetic North Pole, and Earth’s gravity vector $\overrightarrow{g}$ pointing vertically downwards

Data from eleven recording sessions of three hours each were collected on ten adult meerkats at the Kalahari Meerkat Project, as described in [10]; one of the individuals was recorded twice. The individuals bore collars equipped with an inertial measurement unit (IMU) (adapted version of Physilog IV, GaitUp SA, Switzerland) containing a triaxial accelerometer (recording at 100 Hz/axis) and triaxial magnetometer [23], the latter recording at a sampling frequency of 50 Hz/axis with a range of ±1000 μT and 16-bit resolution. The size of the collar case (IMU and battery) was 35 mm × 29 mm × 19 mm, and overall weight was < 25 g. The total geomagnetic field intensity at the study site was 27.3 μT, with a declination angle of 17.9° pointing westwards and a dip (or inclination) angle of 65° pointing upwards, according to the International Geomagnetic Reference Field ([24]; values calculated from https://www.ngdc.noaa.gov/geomag/calculators/magcalc.shtml#igrfwmm). Collars were positioned on the animals so that the axes of the magnetometer were oriented as shown in Fig. 2. The magnetometer was calibrated prior to each recording session according to the method by [25]. The software used to read magnetometer data resampled the data to 100 Hz/axis using linear interpolation (with the 'interp1' function in MATLAB R2016b) to match the sampling frequency of the accelerometer also present on board the recording device.

After the captured animal was collared and released, it was filmed using a handheld video camera recording at 25 frames/second that was synchronised with the collar sensor (see Appendix S1, Additional file 1 for more details). All videos were annotated using Solomon Coder (version: beta 17.03.22). This video annotation served as the groundtruthing data for our behaviour recognition scheme. Archetypal behaviours observed across a wide range of species [10] – foraging, running, and resting – were considered for the ethogram. In addition, we also considered vigilance, a behaviour typical for meerkats, where the individual is stationary and lifts its head and torso to survey its surroundings. Biologically significant information may be derived from these four behaviours [10]: (1) general stress or alertness level through vigilance, (2) periods of inactivity, mainly due to fatigue or excessive heat, through resting (3) proxies for body condition through foraging, and (4) high energy expenditure and possible important events such as aggressive interactions with rival groups through running. Any behaviour dependent upon contextual information, such as territory marking or dyadic social interactions, was excluded from the ethogram.

### Developing candidate features to quantify biomechanical descriptors of movement

Raw triaxial magnetic field intensity data were calibrated and summarised in the form of features quantifying posture, movement intensity, and periodicity. Feature development followed from previous work done with accelerometers [10]. We computed features on a sliding window *w* of size two seconds with an overlap of 50% between successive windows. Windows containing data from exactly one video-labelled behaviour were retained, and those containing transitions between different behaviours were excluded. For each biomechanical descriptor, the candidate features (Table 1) were computed on each two-second window *w* containing *N* = 200 calibrated triaxial magnetic field intensity values recorded along the roll (*m*_*roll*_), pitch (*m*_*pitch*_), and yaw (*m*_*yaw*_) axes.

**Table 1:** Feature development. Candidate features developed to describe the three biomechanical descriptors used in this study: posture (#1), movement intensity (#2 to #5), and movement periodicity (#6 to #9)

S.No.	Biomechanical descriptor	Feature name	Feature description	Computation	
1.	Posture	*meanRoll*	Mean of data from roll axis	$\frac{\Sigma_N{m}_{roll,w}}{N}$	(1)
2.	Intensity	*stdRoll*	Standard deviation of data from roll axis	*std*(*m*_*roll*, *w*_)	(2)
3.		*meanAbsDiffRoll*	Mean of absolute values of time-differentiated roll data	$\frac{\Sigma_N\left|\frac{d}{dt}\left({m}_{roll,w}\right)\right|}{N}$	(3)
4.		*axMaxMeanAbsDiff*	Maximum, across axes, of mean of absolute values of time-differentiated data from each axis	$\underset{\mathrm{A}\in \mathrm{roll},\mathrm{pitch},\mathrm{yaw}}{\max}\left(\frac{\Sigma_N\left|\frac{d}{dt}\left({m}_{A,w}\right)\right|}{N}\right)$	(4)
5.		*avgMeanAbsDiff*	Mean, across axes, of mean of absolute values of time-differentiated data from each axis	$\sum \limits_{A\in roll, pitch, yaw}\frac{\Sigma_N\left|\frac{d}{dt}\left({m}_{A,w}\right)\right|}{3N}$	(5)
6.	Periodicity	*rollFftPeakPower*	Maximum squared coefficient of Fourier transform of data from roll axis	$\underset{\mathrm{i}\in 1\dots \mathrm{L}}{\max}\left({c}_{f_i, roll,w}^2\right)$	(6)
7.		*avgFftPeakPower*	Mean, across axes, of maximum squared coefficient of Fourier transform of data from each axis	$\underset{\mathrm{i}\in 1\dots \mathrm{L}}{\max}\left(\frac{c_{f_i, roll,w}^2+{c}_{f_i, pitch,w}^2+{c}_{f_i, yaw,w}^2}{3}\right)$	(7)
8.		*rollDiffFftPeakPower*	Maximum squared coefficient of Fourier transform of time-differentiated roll data	$\underset{\mathrm{i}\in 1\dots \mathrm{L}}{\max}\left({\delta}_{f_i, roll,w}^2\right)$	(8)
9.		*avgDiffFftPeakPower*	Mean, across axes, of maximum squared coefficient of Fourier transform of time-differentiated data from each axis	$\underset{\mathrm{i}\in 1\dots \mathrm{L}}{\max}\left(\frac{\delta_{f_i, roll,w}^2+{\delta}_{f_i, pitch,w}^2+{\delta}_{f_i, yaw,w}^2}{3}\right)$	(9)

Features were computed on each two-second window *w* containing *N* = 200 calibrated triaxial magnetic field intensity values recorded along the roll (*m*_*roll*_), pitch (*m*_*pitch*_), and yaw (*m*_*yaw*_) axes. Equation numbers are indicated on the right

#### Posture

We obtained a measure of neck inclination with respect to the local magnetic field vector by computing the mean of calibrated magnetic field intensity data recorded in each window *w* along the roll axis (*meanRoll*, equation (1), Table 1).

#### Intensity

We developed four candidate features (#2 to #5, Table 1) to quantify movement intensity: one (*stdRoll*_*w*_, equation (2), Table 1) was aimed at characterising the extent to which *m*_*roll*_ varied in window *w*, whereas the three others aimed to quantify the rate of change of sensor orientation through metrics based on the time-differentiated signal (*meanAbsDiffRoll*_*w*_, equation (3); *axMaxMeanAbsDiff*_*w*_, equation (4); *avgMeanAbsDiff*_*w*_, equation (5), Table 1). Since the differentiation operation results in amplification of sensor- and analog-to-digital signal quantization-generated noise at higher frequencies [26], the raw calibrated magnetometer signal was first low-pass filtered using a Butterworth filter of order 4 and cut-off frequency 10 Hz. MATLAB’s (version R2016b) 'diff' function was used to compute differences between successive signal samples, and each resulting difference was multiplied by the sampling frequency (since, in *d/dt*, *dt* = 1/sampling frequency for discrete signals) to complete the time differentiation operation. To quantify the amount of rate of change in features computed from the time-differentiated signal (features #3, #4, #5 in Table 1), we took the absolute values of each differentiated sample and then computed the mean.

#### Periodicity

We quantified movement periodicity through the use of the Fourier transform (FT). As done in [10], for each window *w*, before computation of the FT, each input signal was filtered with a Butterworth low-pass filter of order 4 and cut-off frequency 10 Hz, normalised, zero-padded to smooth the frequency spectrum [27] by adding 100 zeroes before and after each two-second input signal, and windowed using the Blackman-Harris windowing function. This processed signal was then transformed with a frequency resolution of *U* = 0.01 Hz (corresponding to FT computation at *L* = *Fs*/*U* = 10,000 frequencies), and the squared magnitude of each Fourier coefficient (${c}_{f_i}^2$, *i* ∈ 1…*L*), corresponding to the power of the signal at frequency *f*_*i*_, was computed. Triaxial signals yielded three sets of coefficients, one for each axis: { ${c}_{f_i, roll},{c}_{f_i, pitch},{c}_{f_i, yaw}$ } in the case of the raw calibrated triaxial signal, and { ${\delta}_{f_i, roll},{\delta}_{f_i, pitch},{\delta}_{f_i, yaw}$ } in the case of the time-differentiated signal. For a triaxial signal, the resulting FT was averaged across the three axes. From the final FT, the maximum power obtained across all frequencies *f*_*i*_ (*i* ∈ 1…*L*) was chosen as a measure of the signal periodicity. This FT-based operation was applied to four different input signals in order to develop four candidate features characterising movement periodicity: (1) roll component of the local magnetic field (*rollFftPeakPower*, equation 6, Table 1), (2) triaxial magnetometer signal (*avgFftPeakPower*, equation 7, Table 1), (3) time-differentiated roll signal (*rollDiffFftPeakPower*, equation 8, Table 1), and (4) time-differentiated triaxial signal (*avgDiffFftPeakPower*, equation 8, Table 1). All feature computation was done using MATLAB R2016b.

### Feature selection

To enable direct comparison with the three-feature accelerometer-based model in [10], we selected one feature for each of the three biomechanical descriptors of posture, movement intensity, and periodicity. We tested features quantifying movement intensity (feature # 2 to #5, Table 1) for their efficacy in separating static and dynamic behaviours, and foraging and running. We tested features quantifying movement periodicity (feature #6 to #9, Table 1) for their efficacy in separating foraging and running. We tested five different feature selection methods based on the filter method (using the 'rankfeatures' function in MATLAB R2016b,© 2003–2016 The MathWorks, Inc. See Appendix S3, Additional file 1 for more details) to select one feature to quantify movement intensity, and one to quantify periodicity. *meanRoll* (feature #1, Table 1), being the only candidate developed to describe posture, was chosen by default.

### Behaviour recognition scheme and cross-validation

The behaviour recognition scheme had the same hierarchical tree-like structure and hybrid form as the one found for meerkat behaviour recognition using accelerometers [10]. The scheme consisted of three nodes, each dividing a parent behavioural category (static or dynamic) into two daughter behavioural types (vigilance/resting or foraging/running, respectively). A Support Vector Machine (SVM) was used at each node to obtain optimal feature-value thresholds in a completely automated fashion. At the first node, features encoding information on posture and movement intensity were used to separate static and dynamic behaviours. At the second node, static behaviours were separated into vigilance and resting using postural information. At the third node, dynamic behaviours were separated into foraging and running using information on movement intensity and periodicity. The 'svm' learner in MATLAB R2016b’s 'fitclinear' function (© 2015–2016 The MathWorks, Inc.) was used to train the SVM at each node.

To validate the predictions of the SVM-SVM-SVM hybrid model with the chosen features against groundtruth video-annotated behaviours, two cross-validation methods were tested: (1) stratified ten-fold cross-validation (STRAT), which evaluates model performance when the frequency and duration of different behaviours may be skewed, and (2) leave-one-individual-out cross-validation (LOIO), which evaluates model performance when inter-individual variability is taken into account [10]. We used standard confusion matrix-based metrics to evaluate and compare model performance. These performance statistics included three behaviour-specific metrics (sensitivity, precision, and specificity), and overall model accuracy (see Appendix S2, Additional file 1 for mathematical definitions, computation and interpretation). Custom software was written in MATLAB R2016b to perform cross-validation.

## Results

### Collected data

**Fig. 3:**
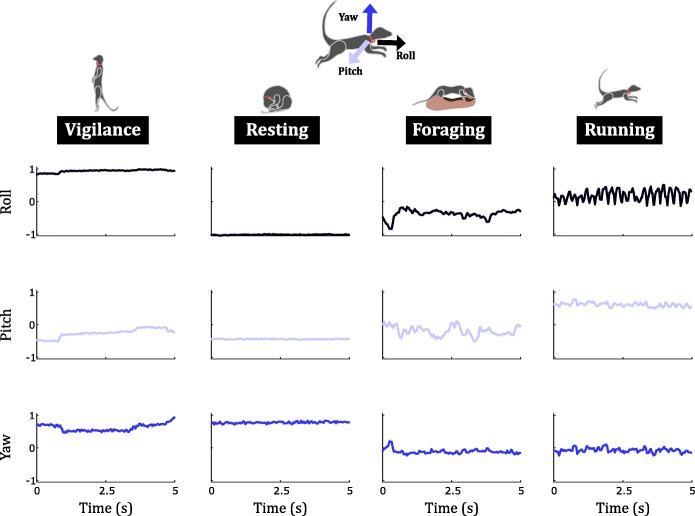
Five-second snapshots of calibrated triaxial magnetometer data for the four behaviours of interest for a typical individual (recording session #1). The horizontal axis shows time in seconds, and the vertical axis represents calibrated, normalised magnetic field intensity measured along the three axes of the sensor in each graph. The signals correspond, from left to right, to bipedal vigilance, curled-up resting, foraging, and running

A total of 82,550 two-second bouts of video-labelled behaviour were collected for the four behaviours of interest (Table 2). The number of bouts collected per animal was 8255 ± 3229 (mean ± SE). The frequency and duration of different behaviours were skewed: foraging (56.2%) was the most common behaviour while running was the rarest (1%). No resting behaviour was observed during six out of the eleven recording sessions; the number of resting bouts collected during the first recording session (55.7% of all resting bouts) far outnumbered those collected during the other recording sessions. Typical signals recorded for the four behaviours (Fig. 3) were found to be in line with our biomechanical hypotheses: static behaviours (bipedal vigilance and curled-up resting) showed little change (Fig. 3, left), while dynamic behaviours (foraging and running) produced greater change in the signals with large, periodic oscillations during running (Fig. 3, right).

**Table 2:** Summary of data collected

Recording Session Number	Vigilance	Resting	Foraging	Running	Bouts per Recording Session
1	4594	2114	1562	69	8339
2	3896	120	5315	29	9360
3	1453	0	6278	38	7769
4	5221	0	2823	161	8205
5	1890	0	6134	169	8193
6	1639	744	4438	98	6919
7	4785	156	3498	40	8479
8	71	0	4841	20	4932
9	4283	0	1713	43	6039
10	1906	0	4407	84	6397
11	1782	661	5398	77	7918
Bouts per Activity	31,520	3795	46,407	828	82,550 (total bouts)

Table adapted from [10]. Triaxial magnetometer data were collected on ten unique individuals; data from recording session #4 and #7 were collected on the same individual. A bout refers to a two-second window *w* containing one video-labelled behaviour

### Features to quantify biomechanical descriptors from triaxial magnetometer data

**Fig. 4:**
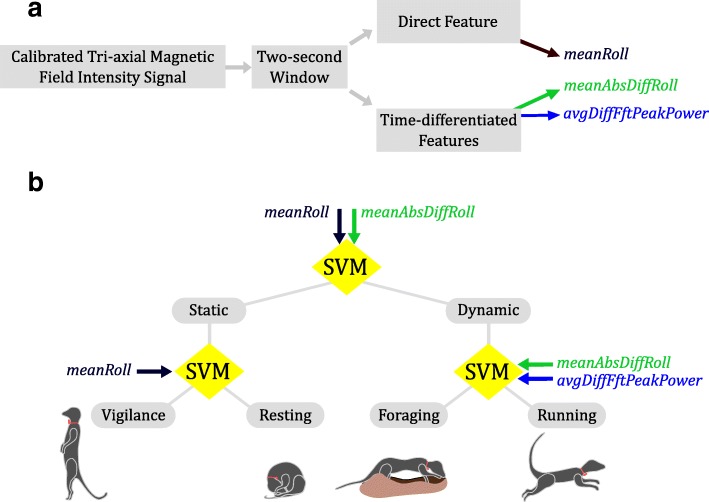
Behaviour Recognition Scheme. (**a**) Flowchart showing feature computation: *meanRoll* quantifies posture, *meanAbsDiffRoll* movement intensity, and *avgDiffFftPeakPower* periodicity. (**b**) Hierarchical classification scheme classifying behaviours as being either static or dynamic, then static behaviours as being either vigilance or resting, and finally dynamic behaviours as being either foraging or running

Measures of posture (*meanRoll,* equation 1, Table 1) and movement intensity (*meanAbsDiffRoll*, equation 3, Table 1) were inputs to the first node to separate static behaviours from dynamic ones (Fig. 4b). Posture (*meanRoll*) was used to distinguish vigilance from resting in the second node, and finally, movement intensity (*meanAbsDiffRoll*) and periodicity (*avgDiffFftPeakPower*, equation 9, Table 1) were used to distinguish foraging from running in the third node (Fig. 4b).

**Fig. 5:**
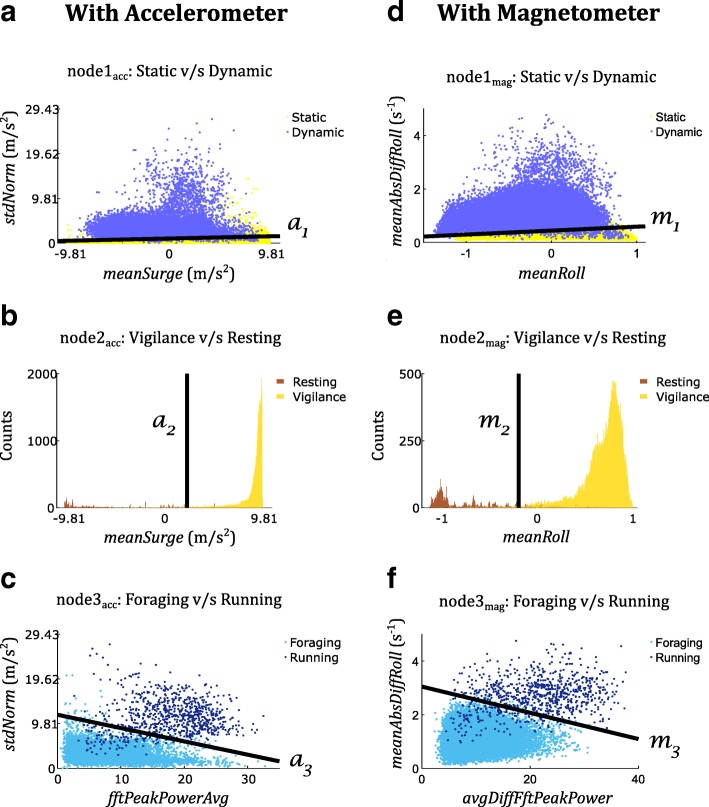
Decision boundaries and feature distributions obtained with accelerometer- (left) and magnetometer-based (right) behaviour recognition with Support Vector Machines trained on the entire dataset for each of the three nodes of the hierarchical behaviour recognition scheme. m_i_ and a_i_ refer to decision boundaries obtained with the magnetometer and accelerometer, respectively, with the subscript i indicating the node index

The use of *meanRoll* to quantify posture produced high separability between bipedal vigilance and curled-up resting (Figs. 3 & 5). During the dynamic behaviours (foraging and running), where the orientation of the animal’s body caused the roll axis of the magnetometer to lie approximately in the horizontal plane, the values recorded along the roll axis (Fig. 3) were in an intermediate range between the extreme positive and extreme negative values recorded during bipedal vigilance (Fig. 1a) and curled-up resting (Fig. 1b), respectively.

Among the features developed to quantify movement intensity, *meanAbsDiffRoll* outperformed the other three candidates with regard to separating both static from dynamic behaviours (Table S2, Appendix S3, Additional file 1), and foraging from running (Table S3, Appendix S3, Additional file 1). Among the features developed to quantify movement periodicity, *avgDiffFftPeakPower* outperformed the other three candidates for the separation of foraging from running (Table S4, Appendix S3, Additional file 1).

### Performance evaluation, and comparison with accelerometer-based behaviour recognition

Magnetometer-based behaviour recognition performance is presented and compared with that achieved with accelerometer data in [10] for STRAT (Table 3) and LOIO (Table 4), and through visual depiction of feature distributions and resulting decision boundaries (Fig. 5).

**Table 3:** STRAT cross-validation results

Sensor	Vigilance			Resting			Foraging			Running			Overall Accuracy (%)
	Sen. (%)	Spec. (%)	Prec. (%)	Sen. (%)	Spec. (%)	Prec. (%)	Sen. (%)	Spec. (%)	Prec. (%)	Sen. (%)	Spec. (%)	Prec. (%)	
Magnetometer	97	98.6	97.8	84.4	99.4	87.1	98.8	97.2	97.8	83.1	99.9	93.9	97.3
Accelerometer	97.1	98.8	98.1	85	99.4	87.1	99.3	97.8	98.3	85.9	99.9	92.1	97.7

The performance of the SVM-SVM-SVM hybrid model with magnetometer data is benchmarked against that obtained with accelerometer data reported in [10]. SVM: Support Vector Machine

**Table 4:** LOIO cross-validation results

Sensor	Vigilance			Resting			Foraging			Running			Overall Accuracy (%)
	Sen. (%)	Spec. (%)	Prec. (%)	Sen. (%)	Spec. (%)	Prec. (%)	Sen. (%)	Spec. (%)	Prec. (%)	Sen. (%)	Spec. (%)	Prec. (%)	
Magnetometer	95.2 ± 2.4	97.9 ± 1.9	95.2 ± 6.2	65.4 ± 25.9	98.9 ± 0.9	77.3 ± 31.1	98.4 ± 0.9	97.0 ± 1.2	95.5 ± 0.5	86.5 ± 3.7	100 ± 0.0	96.4 ± 3.4	96.0 ± 1.5
Accelerometer	95.8 ± 2.8	98.4 ± 1.2	96.4 ± 4.5	71.4 ± 23.6	98.9 ± 1.2	81.1 ± 28.0	98.8 ± 1.0	97.4 ± 1.5	95.3 ± 7.0	86.3 ± 13.2	99.9 ± 0.1	89.1 ± 11.1	96.5 ± 1.8

The performance of the SVM-SVM-SVM hybrid model with magnetometer data is benchmarked against that obtained with accelerometer data reported in [10]. Performance metrics were calculated separately for each test individual, and their mean and standard deviation across test individuals are shown here. SVM: Support Vector Machine

For STRAT, all performance metrics for the most common behaviours (foraging: 56.2% of dataset; vigilance: 38.2% of dataset), and overall model accuracy, were > 95% (Table 3). Good performance was obtained even for the rarer behaviours, resting (4.6% of dataset) and running (1% of dataset), where all behaviour-specific metrics remained > 83%. Further, overall as well as behaviour-wise recognition performance with the magnetometer were similar to that with the accelerometer (Table 3).

For LOIO, data from recording sessions numbers 3, 4, 5, 8, 9 and 10 were discarded since they did not contain any resting behaviour (Table 2). Once again, even when inter-individual variation was taken into account, mean values of all performance metrics for the most common behaviours (foraging and vigilance), and overall model accuracy, were > 95%, and were similar to those obtained with accelerometer-based behaviour recognition (Table 4).

## Discussion

We presented an end-to-end framework to identify common animal behaviours from magnetometer data. Using data collected on 10 wild meerkats, we demonstrated that accurate behaviour recognition can be achieved with a magnetometer alone with performance comparable to that with an accelerometer. Our results shed further light on the magnetometer’s strengths and weaknesses in the context of behaviour telemetry, and suggest possibilities for leveraging the complementary merits of accelerometers and magnetometers within a single classification framework for more robust behaviour recognition.

### Distinguishing dynamic behaviour using magnetometer-derived angular velocity

Differentiating magnetic field intensity with respect to time corresponds to quantifying changes in angles subtended by the Earth’s magnetic field vector onto the three sensor axes with time, and provides an estimate of angular velocity [20]. To separate behaviours based on movement intensity, quantifying change in magnetometer-derived angular velocity was more effective than quantifying change in magnetic field values. This may be because even when the change in sensor inclination angle is small, the rate at which the angle changes may be high. *meanAbsDiffRoll* (equation 3, Table 1) was best at separating static and dynamic, and the two dynamic behaviours. The superior class separability of *meanAbsDiffRoll* implied that using only the roll axis was more effective than when contributions from the other two axes, pitch and yaw, were included. This may have been a consequence of the fact that the roll axis succeeded in capturing both up-and-down, and side-to-side bodily movements made by the meerkat’s neck and torso during dynamic behaviours. The roll axis was also more robust than the other two axes to collar rotations. Magnetic field lines have, in general, a horizontal as well as vertical component – the inclination angle of Earth’s magnetic field at the study site was 65° pointing upwards. The pitch axis would have been insensitive to the up-and-down movements, and the yaw axis insensitive to the side-to-side movements. Further, collar rotations around the meerkat’s cylindrical neck could have confounded class separation through noisy variability in pitch- and yaw-axis contributions for the same activity. Note, however, that the precise choice of the feature describing movement intensity may change when dynamic behaviours of interest involve rotations about the roll axis, such as washing at sea by a Magellanic penguin (*Spheniscus magellanicus*) [5], or fast turning in cheetahs where the weight of the tag causes the collar to rotate around the neck due to centripetal acceleration [28].

Metrics based on magnetometer-derived angular velocity may be better suited than accelerometry for filtering out signal artefacts caused by sensor impacts. Compared to foraging versus running classification using accelerometer data (a3 in Fig. 5c), with the magnetometer there were fewer foraging bouts with low periodicity and high intensity that crossed the decision boundary m3 (Fig. 5f). While exploring the ground for prospective hunting locations, the meerkat’s collar would often bump against vegetation or the ground. Additionally, while digging, the meerkat’s pectoral muscles would hit against the collar. These impacts produced high, transient translational acceleration that led to a higher estimation of bout intensity with the accelerometer. However, the magnetometer, being insensitive to translational acceleration [5], provided a lower estimate for bout intensity due to relatively slow collar orientation changes. Thus, such bouts were correctly classified as foraging with the magnetometer since their intensity placed them below the decision boundary m3 (Fig. 5f). This led to higher precision in the detection of running (7.3% higher mean precision and similar mean sensitivity with LOIO) with much lesser inter-individual variability in performance (9.5% lower standard deviation for sensitivity and 7.7% lower standard deviation for precision) compared to accelerometer-based classification. This was achieved despite running being the rarest behaviour (outnumbered 1:56 by foraging in terms of number of recorded bouts). In a similar fashion, fewer vigilance bouts yielded high enough magnetometer-based intensity to cross over m1 (Fig. 5d) and get misclassified as dynamic behaviour as compared to when the accelerometer (Fig. 5a) was used (Tables S4 & S5, Appendix S4, Additional file 1).

The stringency of the magnetometer in assigning high intensity to a bout of activity was not without its costs. Comparing aggregate confusion matrices observed with the magnetometer and accelerometer (Tables S4 & S5, respectively, Appendix S4, Additional file 1), we observed a higher number of relatively low-intensity foraging bouts getting misclassified as being static, thereby reducing foraging detection sensitivity as compared to accelerometer-based classification (especially for recording sessions #6, #7 and #11, Table S9 in Appendix S4, Additional file 1). This may have been because the amplitude and rate of body movement-generated change in collar orientation during low-intensity foraging behaviour (for instance, during slow ground scratching while keeping the head and torso in the same orientation) may not have been sufficient to generate a large-enough signal detectable above the noise floor introduced by the differentiation operation [26] during computation of *meanAbsDiffRoll*.

Finally, it has been reported that a combination of accelerometers and gyroscopes can lead to better activity recognition in human wearable sensor applications than when each sensor is used alone [20]. In animal studies, the magnetometer may be a viable alternative to the gyroscope for obtaining estimates of angular velocity due to the former’s lower power consumption [29]. This could be important for facilitating long-duration recordings on small animals.

### Estimating posture using magnetometer data

While it was possible to estimate posture using the magnetometer, the accelerometer-based posture measure was nevertheless found to be better at separating static behaviours. In our observations of static behaviour, a number of bouts of quadrupedal vigilance and belly-flat resting were also recorded apart from bipedal or sitting vigilance (Fig.1a), and curled-up resting (Fig. 1b). In these postures, a significant component of the roll axis lay in the horizontal plane. Possibly arbitrary azimuthal orientation of the animal during these postures (Fig.1c) confounded the distinction between quadrupedal vigilance and belly-flat resting. This additional constraint degraded the accuracy of resting detection compared to that with the accelerometer (6% lower mean sensitivity, 3.8% lower mean precision. See also Fig. 5, middle panel). In static behaviours, where the animal’s body retains similar orientation with respect to the horizontal plane, such as during standing and lying in cows (*cf*. [30]), the confounding effect of possibly arbitrary azimuthal orientation may be particularly severe. Further, our implicit assumption that the calibration parameters computed at the beginning of each recording would be valid throughout the recording was found to be only partially true (see Appendix S5, Additional file 1).

### Magnetometer versus accelerometer: similarity and complementarity

Similar behaviour recognition performance with the two sensors suggests that it may not be necessary to make separate considerations for the choice of ethogram when working with magnetometers when archetypal behaviours such as foraging, fast locomotion, and resting are to be identified.

Our results reveal the selectivity of the magnetometer for bodily movement, and relative immunity to signal artefacts arising due to sensor impacts. This may offer the opportunity to study movement energetics using metrics based on magnetometer-derived angular velocity [5], which would be similar but complementary to the acceleration-based metrics ODBA [31] and VeDBA [32]. One advantage of the magnetometer that could be exploited in future studies is the weaker dependence of signal magnitude on sensor location on the animal’s body. When a body segment rotates about a joint, the magnitude of acceleration is higher for distal compared to proximal parts, and this dependence on the location of accelerometer attachment might be especially important to take into account for larger animals. The magnitude of the magnetometer signal during segment rotation, however, would always be the same along a body segment regardless of body size or sensor placement. The apparent pitfall of the accelerometer in confounding bodily movement-produced signals with artefacts arising from sensor impacts could nevertheless be turned to an advantage for other applications where the detection of specific events is desirable. Impact-generated acceleration characteristics have, for instance, been used in the detection of falls in humans [33].

Combining magnetometer and accelerometer data to identify behaviour has been previously suggested [2]. In this study, we develop this idea further and suggest specific aspects of these two sensors to combine for better behavioural identification. Features derived from data from one or both sensors may be chosen according to their specific strengths as inputs for each node of the hierarchical classification scheme (Fig. 4b). For instance, at the first node tasked with separating static behaviours from dynamic ones, the more reliable accelerometer-based posture measure (*meanSurge*) [10] may be combined with the more selective magnetometer-based movement intensity measure (*meanAbsDiffRoll*). Then, *meanSurge* could be used at the second node tasked with separating vigilance from resting on the basis of posture. At the third node, the magnetometer-based intensity (*meanAbsDiffRoll*) and periodicity (*avgDiffFftPeakPower*) metrics may be used for higher-precision distinction between foraging and running. Finally, as has been done for some human movement studies [34], accelerometer and magnetometer data may by combined to give a more accurate and robust three-dimensional estimation of posture in such fused systems than either sensor alone.

## Conclusion

Our findings demonstrate that magnetometers can be used alone to achieve accurate and robust animal behaviour recognition. We showed that sensor tilt with respect to Earth’s magnetic field, and metrics based on magnetometer-derived angular velocity may be used to extract biomechanically significant features to describe posture, movement intensity, and periodicity. Through the directed use of these features in a recently developed hybrid hierarchical behaviour recognition framework combining movement biomechanics and machine learning [10], we found that magnetometer-based behaviour recognition (i) produced similar results to those obtained with the accelerometer, (ii) was robust to inter-behaviour differences in duration and frequency of occurrence, and (iii) exceeded the accelerometer’s resilience to inter-individual variability for dynamic behaviours.

Movements performed by free-living animals, broadly speaking, generate both acceleration as well as angular velocity. Our results reveal that, as long as a sensor can measure a static and dynamic component of movement, key biomechanical descriptors of motion can be quantified and used to recognise common animal behaviours with high accuracy. The generality afforded by the usage of biomechanical considerations to direct inertial sensor data processing, and the simple structure and implementation of the hybrid behaviour recognition framework make it possible to accommodate, compare, and leverage data from accelerometers, magnetometers, and gyroscopes within a single behaviour recognition scheme.

## Additional file

Additional file 1: Five appendices. ‘Appendix S1: Synchronisation of the animal-borne IMU with the hand-held camera’, ‘Appendix S2: Metrics for performance evaluation of classification models’, ‘Appendix S3: Feature Selection’, ‘Appendix S4: LOIO Results’, and ‘Appendix S5: Variation in the Norm of Magnetometer Data’. (DOCX 988 kb)

## Data Availability

Labelled triaxial magnetometer data and feature matrices used to obtain the behaviour recognition results in this study are made available on the Dryad Digital Repository (10.5061/dryad.2fr72sb).

## Abbreviations

FT: Fourier Transform
Hz: Hertz
IMU: Inertial Measurement Unit
KMP: Kalahari Meerkat Project
LOIO: Leave-one-individual-out cross-validation
ODBA: Overall Dynamic Body Acceleration
STRAT: Stratified ten-fold cross-validation
SVM: Support Vector Machine
VeDBA: Vectorial Dynamic Body Acceleration
μT: Micro Tesla
