# Supplementary material for: Behavioural compass: animal behaviour recognition using magnetometers
Source: Mov Ecol. 2019 Aug 27;7:28. doi: 10.1186/s40462-019-0172-6 (PMC6712732; doi:10.1186/s40462-019-0172-6)
Supplement: Supplementary file 1 — Five appendices. ‘Appendix S1: Synchronisation of the animal-borne IMU with the hand-held camera’, ‘Appendix S2: Metrics for performance evaluation of classification models’, ‘Appendix S3: Feature Selection’, ‘Appendix S4: LOIO Results’, and ‘Appendix S5: Variation in the Norm of Magnetometer Data’. (DOCX 988 kb) [file 40462_2019_172_MOESM1_ESM.docx]

**Additional file S1: Synchronisation of the animal-borne IMU with the hand-held camera**

To synchronise videos with the recorded sensor data, a hand-held Physilog IV system (GaitUp SA, Switzerland) connected to an ‘event marker’ was used. The event marker contained a button and an LED that switched on when the button was pressed. Prior to each deployment, the hand-held system was configured to communicate and synchronise with the animal-borne inertial measurement unit (IMU) over a dedicated radio frequency channel. At the beginning and end of each video, we pressed the button on the event marker and filmed the resulting LED. During post-processing, for each video, we matched the moment when the synchronisation radio pulse was received on the animal-borne system with the frame number of the video that showed the event marker’s LED lighting up. Knowing the starting and ending points of the recording in both the IMU and video data enabled us to synchronise both data streams and correct the camera clock’s drift with respect to the IMU micro-processor clock.

**Appendix S2: Metrics for performance evaluation of classification models**

As done in Chakravarty et al. 2019, we used a confusion matrix [$N_{jk}$] to summarise model performance, where $j$, $k$ $\in$ {1: vigilance, 2: resting, 3: foraging, 4: running} and $N_{jk}$ denotes the number of samples of behaviour ‘$j$’ that were predicted by the model to be behaviour ‘$k$’. From this confusion matrix, we computed three behaviour-specific metrics - sensitivity (often referred to as recall in the literature), precision, and specificity, as well as overall model accuracy. These measures are computed as shown in Table S1.

| **Performance statistic** | **Expression** | **Interpretation** |
| --- | --- | --- |
| Sensitivity  (or Recall) | $S_{k}=\frac{N_{kk}}{\Sigma_{i=1}^{4}N_{ki}}$ | How often behaviour $k$ is correctly identified by the model |
| Precision | $P_{k}=\frac{N_{kk}}{\Sigma_{i=1}^{4}N_{ik}}$ | How often the model is right when it predicts behaviour $k$ |
| Specificity | $Sp_{k}=\frac{\Sigma_{i\neq k}\Sigma_{j\neq k}N_{ij}}{\Sigma_{i\neq k}\Sigma_{j=1}^{4}N_{ij}}$ | How often the absence of behaviour $k$ is correctly identified by the model |
| Overall accuracy | $A=\frac{{\Sigma_{i=1}^{4}N}_{ii}}{\Sigma_{i=1}^{4}\Sigma_{j=1}^{4}N_{ij}}$ | How often the model is right, across all behaviours |

Table S1. Measures to quantify model performance, from Chakravarty et al. 2019. Sensitivity (or recall), precision, and specificity are behaviour-specific metrics while overall accuracy summarises overall model performance across the four behaviours

**Appendix S3: Feature Selection**

Feature selection was done using the MATLAB R2016b function rankfeatures (copyright 2003-2016 The MathWorks, Inc.). Five standard filter methods were used for ranking candidate features developed to quantify movement intensity and periodicity, and the results are listed in the sections below.

**1 | Intensity**

This section gives details on feature selection that show how the best feature to quantify movement intensity was chosen from the candidate features for distinguishing between: (i) static versus dynamic behaviour (top node in Figure 4b in manuscript), and (ii) foraging versus running (bottom right node in Figure 4b in manuscript).

**1.1 | Static versus Dynamic Behaviour Classification**

This section compares the candidate features quantifying movement intensity for their efficacy in distinguishing between static and dynamic behaviour.

| Rank | t-test | | Entropy | | Bhatacharyya | | Roc | | Wilcoxon (or Mann-Whitney) | |
| --- | --- | --- | --- | --- | --- | --- | --- | --- | --- | --- |
|  | Feature | Score | Feature | Score | Feature | Score | Feature | Score | Feature | Score |
| 1 | f4 | 336.42 | **f2** | 9.71 | **f2** | 0.08 | **f2** | 0.48 | f1 | 1.41 |
| 2 | **f2** | 489.03 | f4 | 60.88 | f4 | 0.58 | f4 | 0.50 | **f2** | 0.75 |
| 3 | f3 | 390.88 | f3 | 28.13 | f3 | 0.38 | f3 | 0.49 | f4 | 0.01 |
| 4 | f1 | 521.41 | f1 | 37.88 | f1 | 0.41 | f1 | 0.50 | f3 | 0.67 |

Table S2. Ranking features describing movement intensity for static versus dynamic behaviour classification. Serial numbers assigned to features: {f1: *stdRoll*; **f2: *meanAbsDiffRoll***; f3: *axMaxMeanAbsDiff*; f4: *avgMeanAbsDiff*}.

**1.2 | Foraging versus Running Classification**

This section compares the candidate features quantifying movement intensity for their efficacy in distinguishing between the two dynamic behaviours, foraging and running.

| Rank | t-test | | Entropy | | Bhatacharyya | | Roc | | Wilcoxon (or Mann-Whitney) | |
| --- | --- | --- | --- | --- | --- | --- | --- | --- | --- | --- |
|  | Feature | Score | Feature | Score | Feature | Score | Feature | Score | Feature | Score |
| 1 | **f2** | 4.55 | **f2** | 0.55 | **f2** | 0.02 | **f2** | 0.00 | f1 | 1.50 |
| 2 | f3 | 73.24 | f3 | 14.47 | f3 | 0.68 | f3 | 0.49 | **f2** | 0.52 |
| 3 | f4 | 68.34 | f4 | 12.74 | f4 | 0.61 | f4 | 0.48 | f3 | 0.51 |
| 4 | f1 | 62.14 | f1 | 8.93 | f1 | 0.29 | f1 | 0.47 | f4 | 0.47 |

Table S3. Ranking features describing movement intensity for dynamic behaviour classification (foraging versus running). Serial numbers assigned to features: {f1: *stdRoll*; **f2: *meanAbsDiffRoll***; f3: *axMaxMeanAbsDiff*; f4: *avgMeanAbsDiff*}.

**Conclusion**: *meanAbsDiffRoll* consistently outperforms the other three candidate features as a metric to separate both static from dynamic behaviour as well as foraging from running on the basis of movement intensity.

**2 | Periodicity: Foraging versus Running Classification**

This section compares the candidate features quantifying movement periodicity for their efficacy in distinguishing between the two dynamic behaviours, foraging and running.

| Rank | t-test | | Entropy | | Bhatacharyya | | Roc | | Wilcoxon (or Mann-Whitney) | |
| --- | --- | --- | --- | --- | --- | --- | --- | --- | --- | --- |
|  | Feature | Score | Feature | Score | Feature | Score | Feature | Score | Feature | Score |
| 1 | **f4** | 5.30 | **f4** | 0.06 | **f4** | 0.03 | **f4** | 0.04 | **f4** | 0.81 |
| 2 | f3 | 7.85 | f3 | 0.09 | f3 | 0.06 | f3 | 0.07 | f2 | 0.91 |
| 3 | f2 | 42.07 | f2 | 5.04 | f2 | 2.94 | f2 | 0.38 | f1 | 0.80 |
| 4 | f1 | 44.55 | f1 | 9.59 | f1 | 3.16 | f1 | 0.41 | f3 | 0.93 |

Table S4. Ranking features describing movement periodicity for dynamic behaviour classification (foraging versus running). Serial numbers assigned to features: {f1: *rollFftPeakPower*; f2: *avgFftPeakPower*; f3: *rollDiffFftPeakPower*; **f4: *avgDiffFftPeakPower***}.

**Conclusion**: *avgDiffFftPeakPower* consistently outperforms the other three candidate features as a metric for separating foraging from running on the basis of movement periodicity.

**Appendix S4: LOIO Results**

The aggregate and individual-wise confusion matrices obtained with the SVM-SVM-SVM hybrid model during LOIO are provided here.

|  | | Predicted Label | | | |
| --- | --- | --- | --- | --- | --- |
|  |  | *Vigilance* | *Resting* | *Foraging* | *Running* |
| Actual Label | *Vigilance* | **16148** | 227 | 320 | 1 |
|  | *Resting* | 481 | **3208** | 105 | 1 |
|  | *Foraging* | 188 | 107 | **19903** | 13 |
|  | *Running* | 0 | 0 | 47 | **266** |

Table S5. Aggregate confusion matrix obtained during LOIO with magnetometer data. Predictions are arranged along the columns and true labels along the rows; correct predictions are shown in bold.

|  | | Predicted Label | | | |
| --- | --- | --- | --- | --- | --- |
|  |  | *Vigilance* | *Resting* | *Foraging* | *Running* |
| Actual Label | *Vigilance* | **16069** | 279 | 340 | 8 |
|  | *Resting* | 337 | **3252** | 203 | 3 |
|  | *Foraging* | 80 | 102 | **20002** | 27 |
|  | *Running* | 0 | 0 | 47 | **266** |

Table S6. Aggregate confusion matrix obtained with the SVM-SVM-SVM hybrid model for LOIO with accelerometer data, from Chakravarty et al. 2019. Predictions are arranged along the columns and true labels along the rows; correct predictions are shown in bold.

| Vigilance | | | Resting | | | Foraging | | | Running | | | Overall accuracy  (%) |
| --- | --- | --- | --- | --- | --- | --- | --- | --- | --- | --- | --- | --- |
|  | | |  | | |  | | |  | | |  |
| Sen  (%) | Spec  (%) | Prec  (%) | Sen  (%) | Spec  (%) | Prec  (%) | Sen  (%) | Spec  (%) | Prec  (%) | Sen  (%) | Spec  (%) | Prec  (%) |  |
|  |  |  |  |  |  |  |  |  |  |  |  |  |
| 96.7 | 97.2 | 96 | 84.5 | 99.1 | 90.6 | 98.5 | 97.7 | 97.7 | 85 | 100 | 94.7 | 96.4 |

Table S7. Aggregate performance metrics for magnetometer-based behaviour recognition for LOIO using the SVM-SVM-SVM hybrid model.

| Recording Session No. | Vigilance | | | Resting | | | Foraging | | | Running | | | Overall accuracy  (%) |
| --- | --- | --- | --- | --- | --- | --- | --- | --- | --- | --- | --- | --- | --- |
|  | Sen  (%) | Spec  (%) | Prec  (%) | Sen  (%) | Spec  (%) | Prec  (%) | Sen  (%) | Spec  (%) | Prec  (%) | Sen  (%) | Spec  (%) | Prec  (%) |  |
|  |  |  |  |  |  |  |  |  |  |  |  |  |  |
|  |  |  |  |  |  |  |  |  |  |  |  |  |  |
| 1 | 95.6 | 98.6 | 98.9 | 96.3 | 99.0 | 97.1 | 97.1 | 96.8 | 87.3 | 87 | 100 | 96.8 | 96 |
| 2 | 96.2 | 98.5 | 97.9 | 36.7 | 98.1 | 20.2 | 98.8 | 99.2 | 99.4 | 89.7 | 100 | 100 | 96.9 |
| 6 | 92.3 | 99 | 96.5 | 96 | 98.9 | 91.2 | 98.8 | 96.2 | 97.9 | 85.7 | 99.9 | 95.5 | 96.8 |
| 7 | 99.5 | 97.6 | 98.1 | 44.9 | 100 | 97.2 | 98.9 | 98.7 | 98.2 | 82.5 | 100 | 97.1 | 98.2 |
| 11 | 97.3 | 93.6 | 81.6 | 52 | 99.6 | 92.2 | 97.9 | 97.5 | 98.8 | 81.8 | 99.9 | 88.7 | 93.8 |

Table S8. Individual-wise results for magnetometer-based behaviour recognition with the SVM-SVM-SVM hybrid model for LOIO.

| Recording Session No. | Confusion Matrix with Accelerometer | Confusion Matrix with Magnetometer |
| --- | --- | --- |
|  |  |  |
|  |  |  |
| 1 | \| 4310 \| 167 \| 115 \| 2 \| \| --- \| --- \| --- \| --- \| \| 16 \| 1918 \| 179 \| 1 \| \| 18 \| 27 \| 1516 \| 1 \| \| 0 \| 0 \| 19 \| 50 \| | \| 4394 \| 39 \| 161 \| 0 \| \| --- \| --- \| --- \| --- \| \| 28 \| 2036 \| 50 \| 0 \| \| 23 \| 21 \| 1516 \| 2 \| \| 0 \| 0 \| 9 \| 60 \| |
|  |  |  |
| 2 | \| 3834 \| 37 \| 24 \| 1 \| \| --- \| --- \| --- \| --- \| \| 76 \| 44 \| 0 \| 0 \| \| 5 \| 59 \| 5251 \| 0 \| \| 0 \| 0 \| 2 \| 27 \| | \| 3748 \| 118 \| 30 \| 0 \| \| --- \| --- \| --- \| --- \| \| 76 \| 44 \| 0 \| 0 \| \| 6 \| 56 \| 5253 \| 0 \| \| 0 \| 0 \| 3 \| 26 \| |
|  |  |  |
| 6 | \| 1509 \| 69 \| 61 \| 0 \| \| --- \| --- \| --- \| --- \| \| 37 \| 703 \| 4 \| 0 \| \| 1 \| 8 \| 4411 \| 18 \| \| 0 \| 0 \| 3 \| 95 \| | \| 1513 \| 60 \| 66 \| 0 \| \| --- \| --- \| --- \| --- \| \| 16 \| 714 \| 14 \| 0 \| \| 39 \| 9 \| 4386 \| 4 \| \| 0 \| 0 \| 14 \| 84 \| |
|  |  |  |
| 7 | \| 4694 \| 0 \| 86 \| 5 \| \| --- \| --- \| --- \| --- \| \| 41 \| 95 \| 18 \| 2 \| \| 0 \| 5 \| 3485 \| 8 \| \| 0 \| 0 \| 1 \| 39 \| | \| 4759 \| 0 \| 26 \| 0 \| \| --- \| --- \| --- \| --- \| \| 55 \| 70 \| 30 \| 1 \| \| 35 \| 2 \| 3461 \| 0 \| \| 0 \| 0 \| 7 \| 33 \| |
|  |  |  |
| 11 | \| 1722 \| 6 \| 54 \| 0 \| \| --- \| --- \| --- \| --- \| \| 167 \| 492 \| 2 \| 0 \| \| 56 \| 3 \| 5339 \| 0 \| \| 0 \| 0 \| 22 \| 55 \| | \| 1734 \| 10 \| 37 \| 1 \| \| --- \| --- \| --- \| --- \| \| 306 \| 344 \| 11 \| 0 \| \| 85 \| 19 \| 5287 \| 7 \| \| 0 \| 0 \| 14 \| 63 \| |

Table S9. Individual-wise confusion matrices with the SVM-SVM-SVM hybrid model for accelerometer- (left, from Chakravarty et al. 2019) and magnetometer-based (right) behaviour recognition. The entries in each confusion matrix here are arranged according to the row and column headers in Tables S5 and S6.

**Appendix S5: Variation in the Norm of Magnetometer Data**

This section reports the variation in the norm of magnetic field intensity recorded by the animal-borne magnetometer. While the norm of calibrated tri-axial magnetometer data was equal to 1 towards the start of the recording (Figure S1), norm values did not remain at 1 throughout, and varied irregularly during the duration of the recording. This variation in magnetic field intensity is different from the one due to projection of a Cartesian grid on a three-dimensional Earth described by Bidder et al. (2015), since it was observed over the scale of a few metres. We speculate that variation across such short length-scales could only have been caused due to unexpected local magnetic interferences, such as metallic instruments being carried by the person following the animal being recorded (e.g. video-camera, radio receiver, VHF tracking device), the occasional proximity of the animal to fences and power-lines, and the proximity of our study site to the world’s largest land-based manganese field, the base of which is reported to be an iron formation bed formed from ferromagnetic magnetite crystals (Cairncross & Dixon, http://wwwu.edu.uni-klu.ac.at/mmessner/sites/rsa/kuruman/kuruman.htm#f2a). While this seemingly arbitrarily changing magnetic field intensity could have aided the detection of dynamic behaviour because of the coupled effect of changing recorded values due to both movement and external magnetic field intensity change, it could potentially render comparison between postural estimations at different locations difficult. This is because different ranges of recorded values would be obtained depending on the extent of soft-iron and hard-iron effects (Caruso 2000).

Analysis based on all 10,818,151 triaxial magnetometer data points recorded in this study indicates that the overall calibrated magnetometer norm was 1.18 ± 0.13 (mean ± SE) (Table S10).

| Recording Session Number | Mean ± std of calibrated magnetometer norm | Number of tri-axial magnetometer samples |
| --- | --- | --- |
|  |  |  |
| 1 | 0.93 ± 0.35 | 1127349 |
| 2 | 0.82 ± 0.17 | 1111204 |
| 3 | 0.96 ± 0.18 | 1056316 |
| 4 | 0.93 ± 0.14 | 1071018 |
| 5 | 1.26 ± 0.12 | 1039226 |
| 6 | 1.66 ± 0.37 | 1051605 |
| 7 | 1.30 ± 0.33 | 1099111 |
| 8 | 2.09 ± 0.30 | 573149 |
| 9 | 0.81 ± 0.20 | 776537 |
| 10 | 1.50 ± 0.23 | 881126 |
| 11 | 0.72 ± 0.15 | 1031510 |
|  |  |  |
| Mean ± standard error of calibrated magnetometer norm across recording sessions:  **1.18 ± 0.13** | | Total Samples: **10,818,151** |

Table S10. Characterising the variation in calibrated magnetometer norm across recording sessions.

We next demonstrate in Figure S1 the effect of calibration based on Bonnet et al. 2009 by plotting in three-dimensional space the point-clouds obtained pre- and post-calibration. Figure S2 shows a histogram (computed with 200 bins) of calibrated magnetic field intensity norm values for data from all eleven recording sessions, and Figure S3 presents histograms (computed with 200 bins) of recording session-wise data.


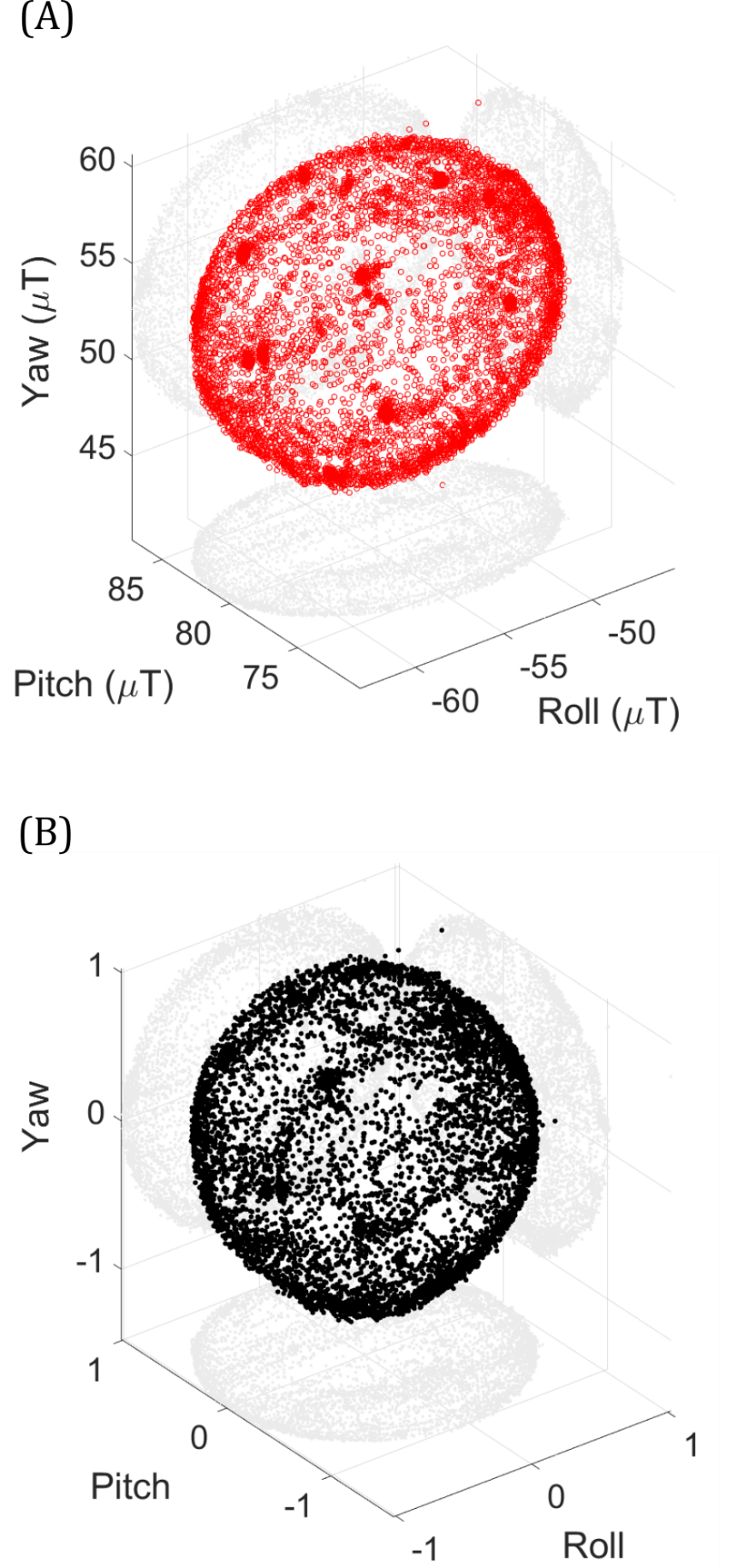


Figure S1. **Magnetometer calibration.**  (A) Uncalibrated data in units of micro-Tesla recorded during the calibration procedure for recording session #10, affected by hard- and soft-iron effects, shown in red hollow circles. (B) The same data after calibration shown in normalised units of magnetic field strength, shown in black solid circles. In both cases, the two-dimensional (2D) projections of the 3D data cluster upon the three mutually perpendicular axis planes are shown in gray circles to permit plane-wise visualisation.


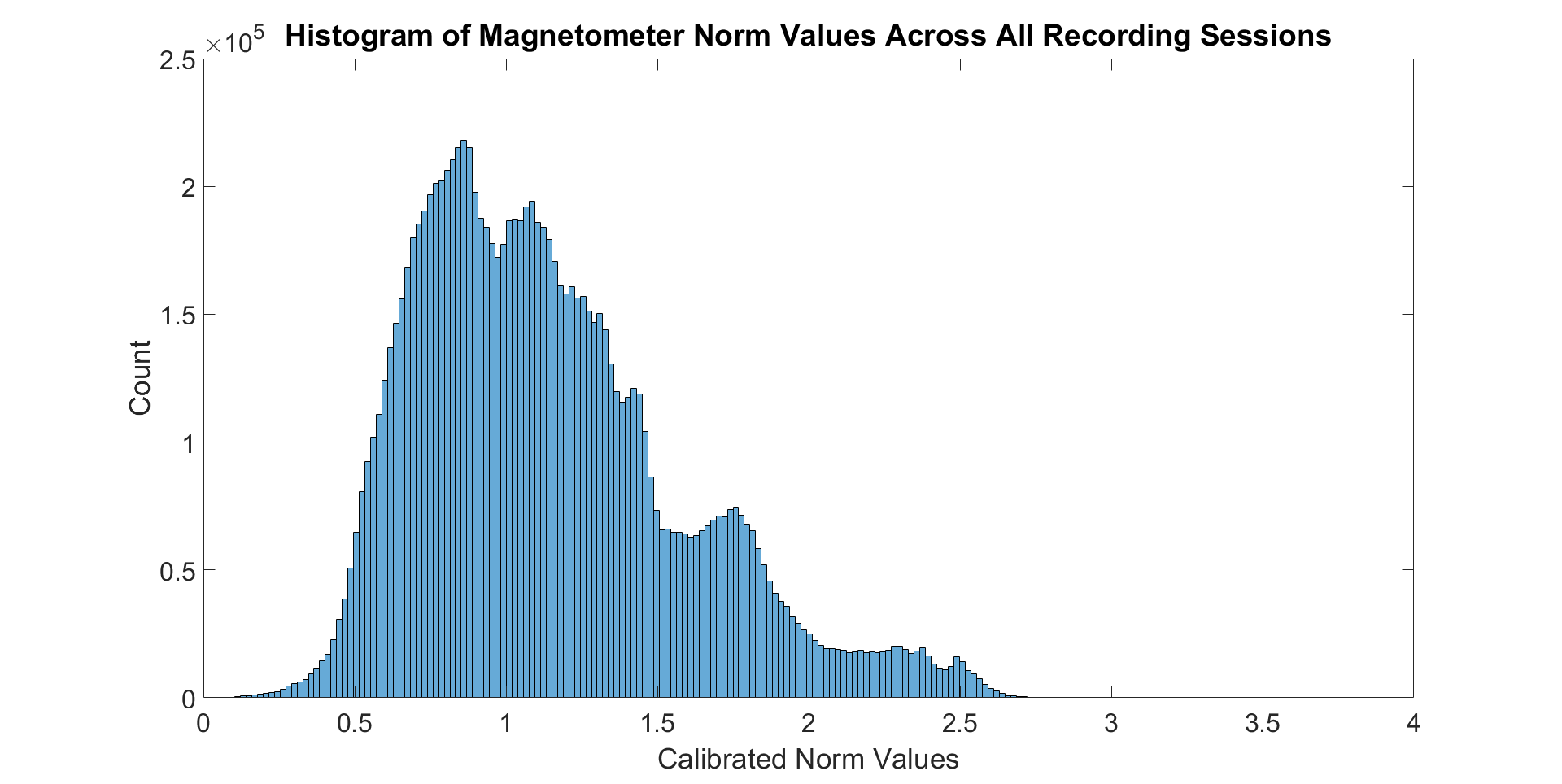


Figure S2. Variation in calibrated magnetometer norm computed across all recording sessions.


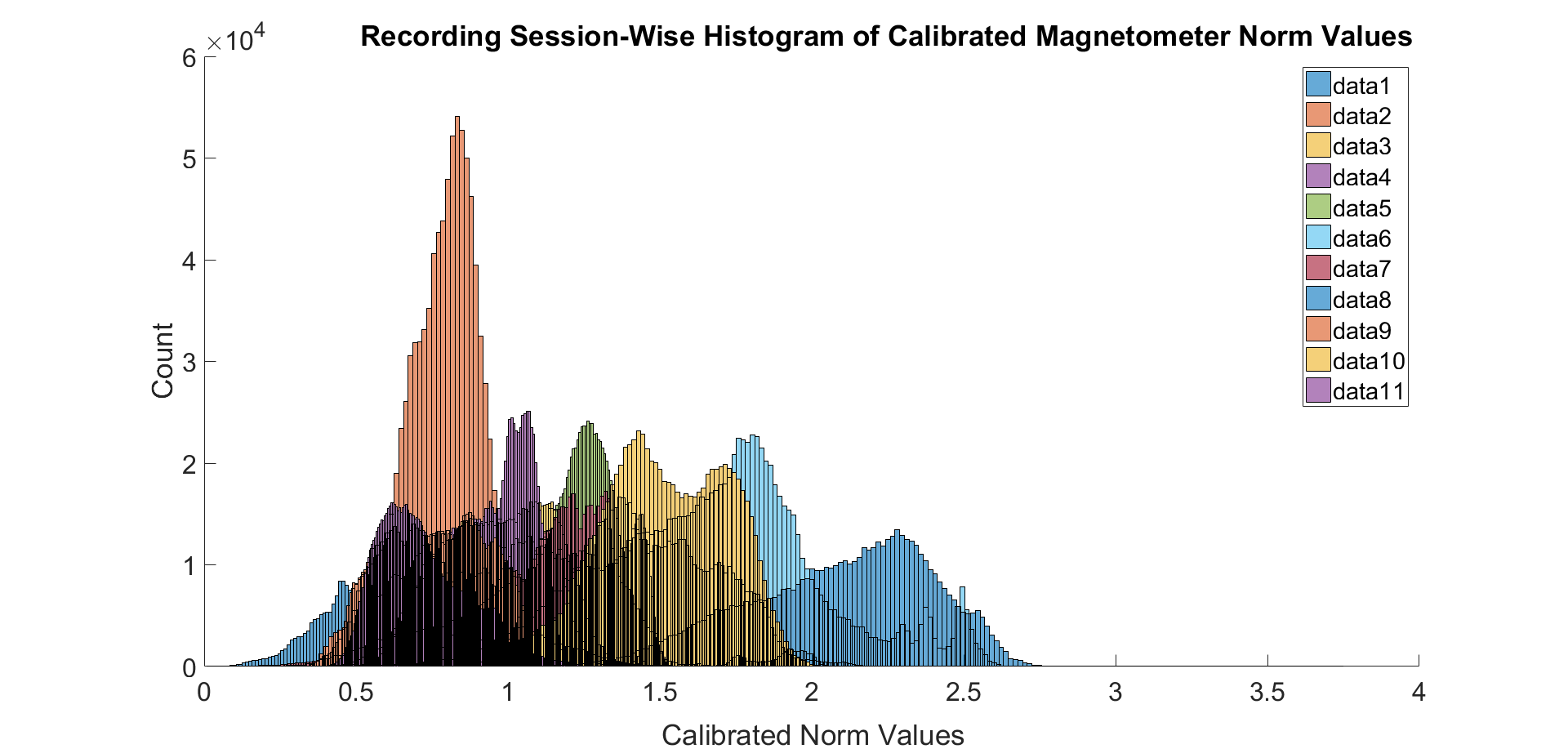


Figure S3. Variation in calibrated magnetometer norm for each of the eleven recording sessions.

**References**

Bidder, O. R., Walker, J. S., Jones, M. W., Holton, M. D., Urge, P., Scantlebury, D. M., ... & Wilson, R. P. (2015). Step by step: reconstruction of terrestrial animal movement paths by dead-reckoning. *Movement ecology*, *3*(1), 23.

Cairncross, B., & Dixon, R. (accessed in March, 2018). Kalahari Manganese Field (Kuruman area) (RSA). <http://wwwu.edu.uni-klu.ac.at/mmessner/sites/rsa/kuruman/kuruman.htm#f2a>.

Caruso, M. J. (2000, March). Applications of magnetic sensors for low cost compass systems. In *Position Location and Navigation Symposium* (pp. 177-184).

Chakravarty, P., Cozzi, G., Ozgul, A., & Aminian, K. (2019). A novel biomechanical approach for animal behaviour recognition using accelerometers. *Methods in Ecology and Evolution*.
